# The Field Metabolic Rate, Water Turnover, and Feeding and Drinking Behavior of a Small Avian Desert Granivore During a Summer Heatwave

**DOI:** 10.3389/fphys.2019.01405

**Published:** 2019-11-20

**Authors:** Christine Elizabeth Cooper, Philip Carew Withers, Laura Leilani Hurley, Simon Charles Griffith

**Affiliations:** ^1^School of Molecular and Life Sciences, Curtin University, Perth, WA, Australia; ^2^Department of Biological Sciences, Macquarie University, Sydney, NSW, Australia; ^3^School of Biological Sciences, University of Western Australia, Perth, WA, Australia; ^4^School of Biological, Earth, and Environmental Sciences, University of New South Wales Sydney, Sydney, NSW, Australia

**Keywords:** bird, climate change, field metabolic rate, field water turnover rate, heatwave, foraging, temperature, zebra finch

## Abstract

Global environmental change is leading to an increase in the frequency, intensity, and duration of extreme weather events, so effective environmental management requires an understanding not only of the physiological response of organisms to increased mean temperatures, but also to extreme environmental conditions. To determine the physiological consequences of heatwaves on energy and water balance of arid-adapted zebra finches (*Taeniopygia guttata*), we measured field metabolic rate and water turnover rate of wild, free-living finches during a heatwave (consecutive days of maximum ambient temperature of 40–45°C) and during a cooler period (maximum ambient temperature of 28°C) during a summer drought. To understand how birds accommodated their energy and water requirements, we also monitored feeding and drinking behavior of zebra finches at the study site on hot and cold days over 2.5 months during the same summer. Zebra finches can accommodate heatwaves without major impacts on field energy or water turnover, even when the heatwave is superimposed on high summer temperatures and long-term drought, so long as drinking water is available. In fact, cooler periods may pose a greater energetic challenge than heatwaves during drought, when food availability is limited, due to the increased thermoregulatory cost of maintaining a high body temperature against a thermal gradient. Zebra finches avoided or limited activity during the most thermally challenging periods of the day. Their pre-emptive feeding and drinking in preparation for hours of relative inactivity at high ambient temperature, together with a high body water content and reduced midday activity and metabolic heat production, enabled zebra finches to maintain body mass during a heatwave. Predicting upcoming periods of unfavorably high ambient temperature, together with a high body water content, may be essential for survival by desert birds of extreme ambient temperature during heatwaves.

## Introduction

Global environmental change is not only impacting average climatic conditions such as mean ambient temperature and annual rainfall but is also increasing the intensity, frequency, and duration of extreme weather events such as heatwaves (Meehl and Tebaldi, 2004; Tebaldi et al., 2006; Diffenbaugh and Field, 2013; IPCC, 2014). These extreme weather events may have a significant, immediate, and long-lasting impact on biodiversity, especially when combined with an underlying gradual mean change in climate (Saunders et al., 2011; Harris et al., 2018; Ruthrof et al., 2018). How animals respond to extreme environmental conditions provides exemplary insights into animal function as it is under these conditions that physiological processes must be most highly refined for survival. Therefore studying species during these extreme events will provide essential basic information about the physiological ecology of species. In addition, attempts to limit biodiversity loss by managing species in these changing conditions require a detailed mechanistic understanding of the physiological response of organisms to environmental variability (Harris et al., 2018; Conradie et al., 2019). However, our current understanding of the physiological function of most organisms is inadequate to determine effective mitigation strategies for even background climate change, and is even less able to predict and manage their response to unpredictable, extreme weather events (Chown et al., 2010; Ratnayake et al., 2019).

Birds are particularly susceptible to extreme weather events (Albright et al., 2017). They have a small body mass, are generally diurnally active and restricted to surface microhabitats, have high mass-specific energy and water requirements and a high body temperature (*T*_b_) that is close to the lethal maximum for endotherms (Kendeigh, 1969; Withers et al., 2004; Williams and Tieleman, 2005). Mass mortality as a consequence of extreme heatwaves has been reported for various avian species, even iconic desert species such as budgerigars (*Melopsittacus undulatus*) and zebra finches (*Taeniopygia guttata*; Finlayson, 1932; McKechnie and Wolf, 2010; Saunders et al., 2011; McKechnie et al., 2012; Birdlife Australia, 2014), which inhabit naturally harsh, unpredictable arid environments. If these mortality events impact on uncommon or range-restricted species, or occur at a sufficiently large scale or frequency, they can have substantial impacts on the long-term viability of populations and even species (Saunders et al., 2011; Franklin et al., 2014; Ruthrof et al., 2018). For example, a sub-continental heatwave in Western Australia during 2011 was implicated in wide-spread abrupt and synchronized biotic effects in marine and terrestrial environments (Ruthrof et al., 2018), including reduced chick production and increased mortality of breeding little penguins (*Eudyptula minor*; Cannell et al., 2012). On a single day of extreme high temperature in southern Western Australia during 2010, at least 208 endangered Carnaby’s cockatoos (*Calyptorhynchus latirostris*) were killed, with likely further deaths of orphaned chicks (Saunders et al., 2011).

During extreme heatwaves, terrestrial birds are impacted by the immediate trade-off between maintaining *T*_b_ at sub-lethal levels, typically <46°C (Kendeigh, 1969; Withers et al., 2004; Conradie et al., 2019; McKechnie and Wolf, 2019), using evaporative cooling, and fatal dehydration, generally assumed to be 11–15% body mass loss (Wolf, 2000; Albright et al., 2017). Birds can manipulate this trade-off by integration of physiological and behavioral responses, including reduction in activity and retreat to shaded, cooler microclimates, facultative hyperthermia, enhanced evaporative heat loss, and heat dissipation behaviors such as wing drooping and panting/gular fluttering (Wolf, 2000). However, these responses may affect a bird’s ability to replenish body water by drinking, and may also hamper maintaining energy balance, due to reduced foraging opportunities and efficiency (Smit et al., 2016; Conradie et al., 2019; Funghi et al., 2019). For example, southern pied babblers (*Turdoides bicolor*) were unable to maintain body mass on days with maximum ambient temperature (*T*_a_) >35.5°C due to the impact of heat dissipation behavior on foraging efficiency (du Plessis et al., 2012). Balancing energy and especially water budgets with fatal hyperthermia is especially challenging for small birds during periods of extreme heat compared to larger species, as they have a smaller body water pool and energy store and consequently a shorter survival window, coupled with higher rates of environmental heat gain and high mass-specific evaporative water loss (EWL) and energy use (Smit et al., 2016).

Here we examine how short periods of extreme temperature, superimposed over longer term conditions of drought and high summer temperature, impact on the energy and water turnover of wild, free-living zebra finches in an arid habitat in central Australia. Zebra finches are small (10–13 g), granivorous, estrildid finches that inhabit much of the Australian arid zone. They are a widely used model for investigating the physiological and behavioral mechanisms by which desert birds survive and reproduce in desert environments (Zann, 1996). We measure field metabolic rate (FMR) and water turnover rate (WTR) of free-living birds during periods of extremely high *T*_a_ (>40°C) when maximum *T*_a_ exceeds the average diurnal passerine *T*_b_ (McKechnie et al., 2012; Albright et al., 2017), and comparatively milder *T*_a_ (maximum *T*_a_ ≤ 28°C). We compare these rates of energy and water turnover with diurnal patterns of foraging and drinking behavior, to provide information regarding the physiological and behavioral mechanisms that determine how extreme weather events may impact the survival and of small desert birds (McKechnie et al., 2012). We hypothesize that extreme heatwave conditions will reduce foraging opportunities, with a negative impact on energy turnover and significant body mass loss, and that water turnover and drinking rates will increase, during hot compared to cooler conditions.

## Methods

Zebra finches were studied at Gap Hills (31°05′ S, 142°42′ E), Fowlers Gap Arid Zone Research Station, approximately 112 km north of Broken Hill, New South Wales, Australia. The study occurred during December, January, and February 2018, within a 2-year period of extended drought and above-average temperatures (Australian Bureau of Meteorology, 2018). Four feeders, consisting of a 50 cm × 30 cm seed tray of commercial finch seed placed inside a 70 cm × 40 cm × 50 cm wire cage with a drop door, were opened at the site approximately 2 months before the study. The only free water known to be available within at least 5 km of the empty dam at Gap Hills was provided at two small drinking troughs (about 800 m and 100 m from the dry dam), filled from enclosed plastic water tanks with a float valve, and protected from large mammals and emus by a mesh fence. In the 50 months leading up to the fieldwork, a total of 771 finches had been captured at the site and were tagged subcutaneously with a passive integrated transponder (PIT; Minichip; Micro Products Australia, Perth, Australia), as part of other studies (e.g., Brandl et al., 2018; Funghi et al., 2019). All individuals were also banded with an individually numbered metal leg band (size 02) supplied by the Australian Bird and Bat Banding Scheme (ABBBS). Mark-recapture estimates suggest that approximately 350 individual birds were resident at the site during the study (Cooper, personal observation), but these were not all PIT tagged.

Field metabolic rate and WTR were estimated using the doubly labeled water method (Nagy, 1983; Speakman, 1997) to measure rates of CO_2_ production and water flux for 10 wild, free-living zebra finches during a period of four consecutive days (18–21 January 2018) where maximum ambient temperature for the station exceeded 40°C (range 40–44.5°C; hot period), and for 10 finches during a period of 2 days (1–2 February 2018) when maximum ambient temperature was 27–27.6°C (cool period). The maximum temperature anomaly (deviation from the 30-year monthly average during 1961–1990; Australian Bureau of Meteorology) for these periods ranged from 4.4 to 8.3°C and −6.9 to −7.7°C respectively.

During the FMR and WTR measurements, only one feeder (and the two water tanks) remained open. A vehicle was used as a hide near the feeder, and finches were observed with binoculars and captured by the observer dropping the feeder door using a string trigger. Twenty finches were initially captured shortly after sunrise of the first day of the hot or cool period respectively. They were weighed to 0.1 g on an electronic balance, banded with both a uniquely numbered ABBBS metal band and a plastic color band, and given an IP injection of ~0.057 ml of water consisting of a 2:1 ratio of 98% enriched ^18^O and ^2^H; injection syringes were weighed before and after, to 0.0001 g. Finches were held in a cloth bag for approximately an hour (mean 1 h 9 min), after which an equilibration blood sample (~75 μl) was taken from the brachial vein before they were released. Ten finches in each experimental period were recaptured 18.7–71 h later by observing color-banded birds entering the feeder and triggering the drop door, and a recapture blood sample (~75 μl) was immediately taken. Blood samples (~75 μl) from six additional finches not involved in the turnover study were taken for analysis of background level of isotopes.

Blood samples were immediately flame-sealed on-site in the heparinized glass capillary tubes in which they were collected, using a butane burner. They were returned to the laboratory at the University of Western Australia, where water was extracted by vacuum distillation in flint glass pipettes. The enrichment of the injectate was calculated by diluting 0.061 g of the isotope solution into 7.56 g of water taken from the water troughs at the site. The solution was thoroughly mixed and samples before (background) and after (equilibration) addition of the injectate were also vacuum distilled and analyzed.

The stable isotope composition of distilled samples was analyzed by the West Australian Geochemistry Centre at the University of Western Australia (Skrzypek and Ford, 2014), using a Picarro L1115-I isotopic liquid water analyzer (Picarro, Santa Clara, California, USA). Prior to analyses, all samples were isotopically diluted with deionized water with a known isotope composition. The *δ*^2^H and *δ*^18^O raw values were normalized to the Vienna Standard Mean Ocean Water (VSMOW) scale after Skrzypek (2013), with three-point normalization replicated twice and calibrated against international standards provided by the International Atomic Energy Agency (Coplen, 1996). Body water content (BWC), water flux, and CO_2_ production were calculated from these values (converted to ppm), after Speakman (1997). WTR was calculated as r_H2O_ = N_H_k_D_, where r_H2O_ is the H_2_O turnover rate (mol h^−1^), N_H_ is the average ^2^H dilution space (mol), and k_D_ is the ^2^H turnover (h^−1^). FMR was calculated using Speakman’s (1997) revision (i) of the Lifson and McClintock (1966) method as r_CO2_ = N_O_(0.48123k_O_ − 0.48743k_D_), where r_CO2_ is the CO_2_ turnover rate (mol h^−1^), N_O_ is the average ^18^O dilution space, and k_O_ is the ^18^O turnover (h^−1^).

The mean of the six background values was used in calculations for all birds. For five birds, for which equilibration samples could not be analyzed, the mean value for the equilibration samples of the other 15 birds was used. Total BWC was calculated from the ^18^O dilution. FMR was converted from ml CO_2_ day^−1^ to kJ day^−1^, assuming 25 kJ L^−1^ CO_2_ (Nagy et al., 1999). Metabolic water production (MWP) was calculated (based on a millet seed diet) as 0.62 mg ml^−1^ O_2_ consumed (MacMillen and Hinds, 1983; MacMillen and Baudinette, 1993) and an oxyequivalence of 20.3 ml O_2_ kJ^−1^ (Withers et al., 2016).

Feeding and drinking behavior was assessed from 1 December to 19 February (excluding the days on which FMR and WTR were measured) by monitoring birds accessing the feeders and water troughs using an 11-cm diameter antenna connected to a PIT tag reader (RFIDRW-E-232; Priority 1 Design, Melbourne, Australia) that recorded a tagged bird’s unique ID code every time it passed through the antenna, with the date and time. Feeders were topped up with seed and water troughs checked daily, and the PIT recorder and timing checked (by passing a PIT through the antenna at a known time) and data downloaded every 1–5 days. Feeding behavior was analyzed for the four feeders on seven cool days when the maximum *T*_a_ was <30°C (mean maximum 25.6 ± 0.95°C, maximum range 22.2–29.8°C) and 17 hot days when the maximum *T*_a_ was >40°C (mean maximum 42.5 ± 0.42°C, maximum range 40.1–45.2°C), and for the water troughs for 26 hot and nine cool days, within 7 weeks of the FMR/WTR studies. The total number of times a bird passed through the antennae was summed for the four feeders and summarized into 30-min bin ranges, as were the data for the two water troughs.

Values are presented as mean ± S.E. with *N* = number of individuals, and times are Australian Central Summer Time (ACST; GMT + 10.5 h). Statistical analyses were accomplished with *statisti*XL (www.statistiXL.com). Body water content, FMR, and WTR were compared during hot and cool periods using two sample *t*-tests with a test, and if necessary correction, for equality of variance. Frequency of visits to the feeders and water troughs (analyzed separately) throughout the day was compared for hot and cool periods using a two-way contingency table (hot/cool vs. time of day). The frequency distribution of visits to feeders and water troughs during hot periods was directly compared to that during cool periods by using the distribution during cool periods as the expected values (adjusted to the same total number of visits during hot periods).

## Results

Birds captured during hot periods had a higher body mass (12.2 ± 0.20 g, *N* = 20) than during cool periods (11.5 ± 0.17 g, *N* = 20; *t*_38_ = 2.43; *p* = 0.020), but body mass did not differ significantly for those 10 individuals that were recaptured during each period and for which FMR and WTR were measured (12.1 ± 0.23 hot and 11.4 ± 0.21 cool, mean = 11.7 ± 0.17 g, *N* = 20; *t*_18_ = 2.09; *p* = 0.051). There was no difference in the body mass change between initial capture and recapture for hot and cool periods (mean = 0.06 ± 0.116 g; *t*_18_ = 0.966, *p* = 0.347).

Enrichment of ^18^O and ^2^H for recaptured birds was at least 268 and 216 ppm higher than the mean background value respectively, and exceeded the maximum measured for background samples by more than three (^18^O) and seven (^2^H) times. Body water content was 75.4 ± 2.14% (*N* = 20) and WTR was 4.5 ± 0.30 ml day^−1^ (*N* = 20), and neither differed between hot and cool periods (*t*_18_ ≤ 1.73; *p* ≥ 0.187). Field metabolic rate was significantly lower (*t*_18_ = 3.54, *p* = 0.002) during hot (29.4 ± 2.41 kJ day^−1^) compared with cool periods (44.0 ± 3.35 kJ day^−1^). As a consequence, MWP calculated from FMR was correspondingly lower during hot (0.953 ± 0.092 ml day^−1^) compared to cool periods (1.317 ± 1.00 ml day^−1^), and was 20 ± 2.3 and 34 ± 2.6% of the birds’ WTR respectively.

**Figure 1:**
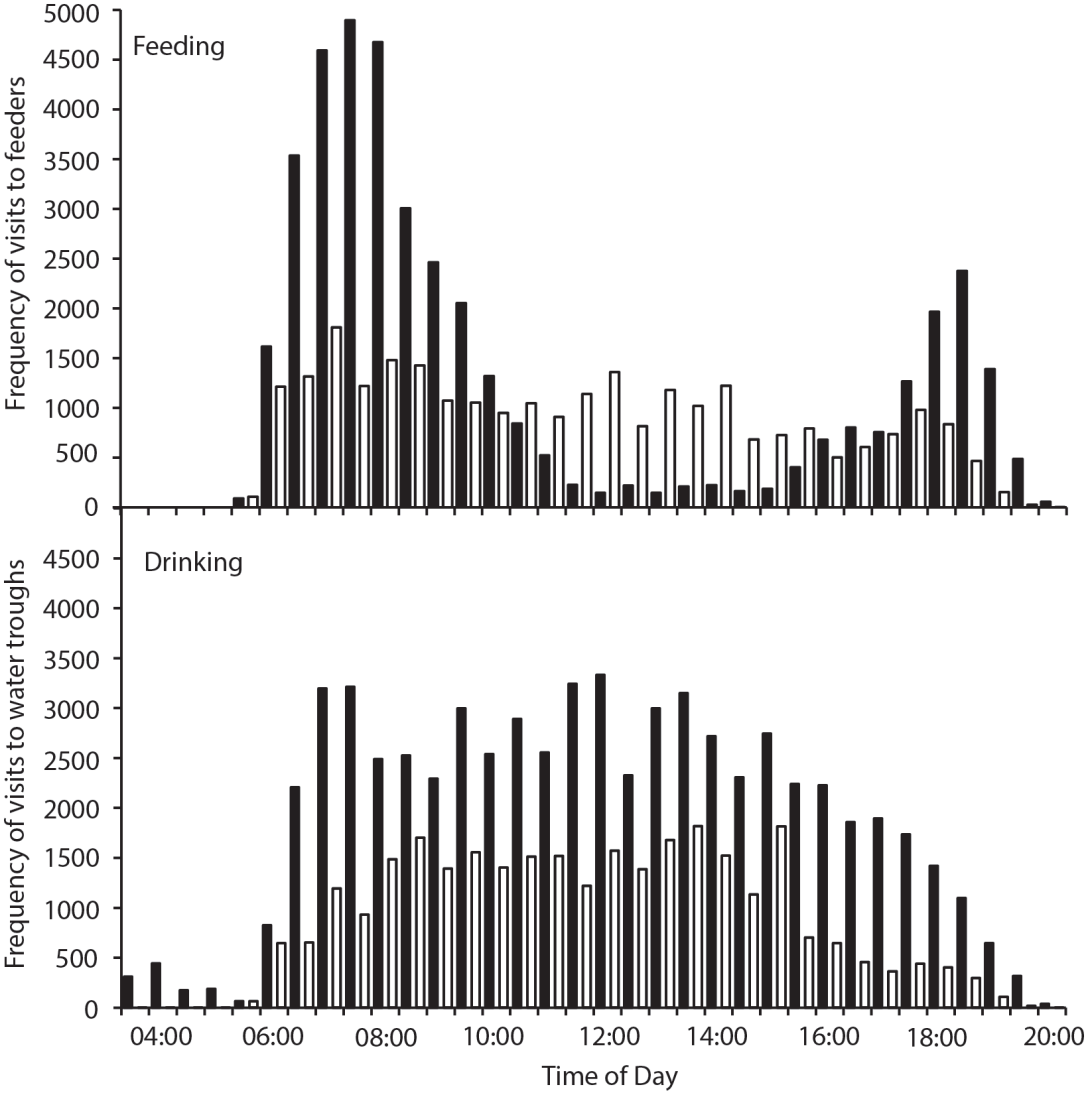
Frequency of visits to four feeders (top panel) and two water tanks (bottom panel) by zebra finches (*Taeniopygia guttata*) in an arid habitat during hot periods (maximum daily *T*_a_ > 40°C; black bars) and cool periods (maximum daily *T*_a_ < 30°C; white bars).

**Figure 2:**
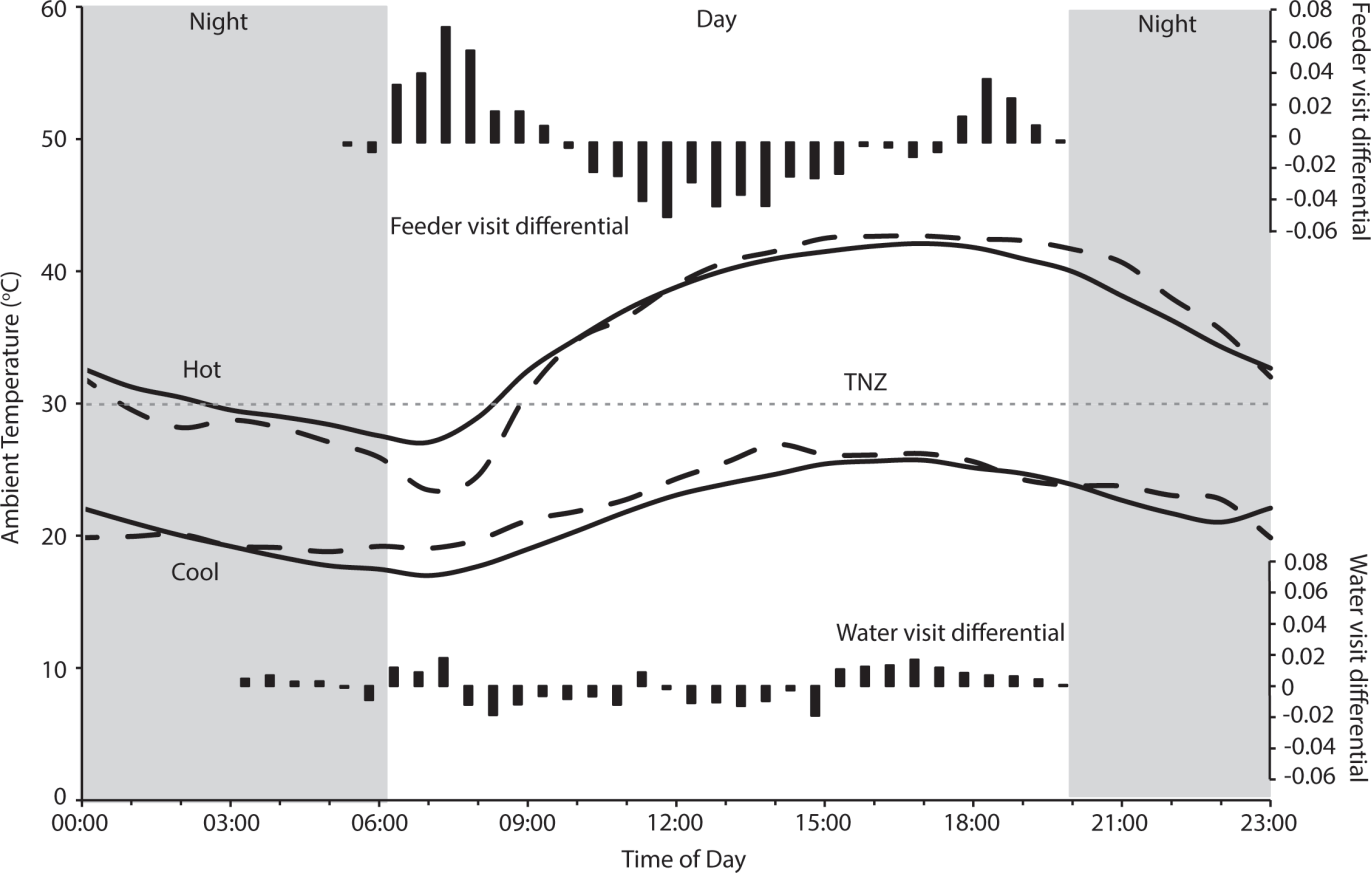
Hourly temperature during hot and cool periods at Fowlers Gap research station, when visits to feeders and water troughs by zebra finches (*Taeniopygia guttata*) were monitored (solid lines), and when FMR and WTR were measured (dashed lines). The gray dashed line indicates thermoneutrality (TNZ; 30°C) for zebra finches (Calder, 1964; Cade et al., 1965) and the gray panels indicate the period from sunset to sunrise. Black bars indicate the differential of frequencies (from Figure 1) of visits on hot to cool days, standardized to total number of hot day visits, to four feeders (top panel) and two water tanks (bottom panel). Temperature data sourced from the Australian Bureau of Meteorology for Fowlers Gap (station number 046128).

There were 236 individual birds recorded visiting feeders, and 252 individuals recorded visiting water troughs during the study period. Birds visited the feeders between 05:30 and 20:00, and the water tanks between 03:30 and 20:00, with between 707 and 7,838 total visits to the feeders, and 116–13,182 total visits to the water troughs recorded per day. Two-way contingency tables indicated a highly significant interaction between hourly visitation frequency and hot and cool periods for both feeding and drinking ($\chi_{29}^{2}$ = 13,310, *p* < 0.001; $\chi_{33}^{2}$ = 2,817, *p* < 0.001 respectively; Figure 1), and the frequency of feeding and drinking activity throughout the day was significantly different during hot (predominantly morning and evening activity) and cool (more uniform over the daytime) periods ($\chi_{33}^{2}$ = 38,433, *p* < 0.001; $\chi_{33}^{2}$ = 172,814, *p* < 0.001, respectively; Figure 2).

## Discussion

The wild, free-living zebra finches that we studied at Fowlers Gap are iconic desert birds, withstanding short periods of extreme temperature in their harsh, arid-zone environment. A period of 3–4 days with maximum *T*_a_ > 40°C had minimal impact on the energy and water balance of the zebra finches. A combination of high BWC, a granivorous diet (with high digestibility that reduces foraging requirements), and the ability to behaviorally adjust foraging times in anticipation of high daytime environmental temperatures allows zebra finches to maintain body mass and meet their energy and water requirements when *T*_a_ exceeds thermoneutraltity for the majority of the day. Our data suggest that high *T*_a_ is actually less of an energetic stress than cooler days, and zebra finches are able to thermoregulate within tolerable limits without excessive water use, at least when maximum *T*_a_ does not exceed 45°C. Our results provide insight into the mechanisms by which birds can withstand extreme temperatures, and highlight situations when extreme environments may become lethal to birds.

### Body Water Content

The BWC of birds is typically about 63–66% (Hughes et al., 1987; Ellis and Jehl, 1991; Speakman, 1997), although values of 70–80% have been reported, particularly for arid-habitat birds (e.g. Ambrose et al., 1996; Anava et al., 2000). Our values for zebra finches were relatively high (75%), and higher than for captive zebra finches (63%; Skadhauge and Bradshaw, 1974), and may indicate poor body condition with a low fat content (Johnson et al., 1985; Hughes et al., 1987; Ellis and Jehl, 1991). This presumably reflects the long-term drought and limited availability of food in an already harsh arid environment. This inference is supported by the observation that there had been little to no breeding activity of these birds in artificial next boxes (which generally support breeding, e.g., Griffith et al., 2008; Brandl et al., 2018) at the site during the previous year (Hurley and Griffith, personal observation). Although BWC can be overestimated if the period between injection of the isotope and sampling of blood for the equilibration sample is too long, our equilibration time of 1 h is typical for small birds (Speakman, 1997). A MWP of 1.317 ml day^−1^ (during cool periods when MWP was highest) would have produced only 0.055 ml of water during the 1-h equilibration period, which cannot account for the “extra” 1.028 ml of body water of a zebra finch with a calculated BWC of 75% compared to 66%. Body water content can also be overestimated if the injection volume was overestimated, for example by leakage from the injection site, or loss of injection solution from the syringe after pre-injection weighing. We cannot unequivocally eliminate this as a source of error, but did not observe leakage or spillage of injection solution. We therefore consider that our high value for BWC of zebra finches is real, and likely adaptive (see below).

### Field Metabolic Rate and Water Turnover Rate

Our measures of FMR and WTR are consistent with allometric predictions for desert birds, and generally consistent with observations from captive individuals. Field WTRs of our zebra finches (4.5 ml day^−1^) were 99–113% of that predicted for desert birds, and 49–57% of that for all birds, after Tieleman and Williams (2000), and 80–92% and 74–85% respectively after Nagy and Peterson (1988), reflecting the more frugal water turnover of birds in arid habitats. The WTR we measured for wild, free-living zebra finches was also within the range of water intake rates of 3.8–5.4 ml water day^−1^ measured for captive zebra finches (Skadhauge and Bradshaw, 1974). The small body size and therefore relatively high metabolic rate of small birds means that they can meet a considerable proportion of their daily water requirements *via* MWP (Bartholomew, 1972; Bradshaw, 2003). For wild, free-living finches in an arid habitat in summer, MWP accounted for 20–34% of WTR, which was consistent with the 31.3% of daily water flux of zebra finches in the laboratory (Skadhauge and Bradshaw, 1974).

The extremely hot conditions during our FMR measurements did not negatively impact the zebra finches’ energy use, with finches maintaining body mass over the hot period. We calculated 1.9–2.9 g fresh matter intake day^−1^ for our finches (from FMR for a granivore after Nagy, 2001), which is 63–90% of that predicted for a passerine bird and 32–46% of that predicted for a desert bird, reflecting the high metabolizable energy content, and therefore low fresh matter intake, of a granivorous diet (Nagy, 2001). The FMR that we measured for our birds during this study was 2.0–2.8 times BMR for this species (Rønning et al., 2005; Cooper et al., in review), consistent with the generalization that FMR is 2–3 times BMR (Degen and Kam, 1995; but see Koteja, 1991 and Ricklefs et al., 1996).

### Feeding and Drinking

On days of extremely high *T*_a_, zebra finches dramatically reduced foraging, and to a lesser extent drinking, during the hottest part of the day, as is typical for birds experiencing extreme heat and solar radiation (Wolf, 2000). Activity for foraging and drinking will increase metabolic heat production, and doing so in unshaded locations typical of arid environments also exacerbates an already high *T*_a_ with high incident solar radiation (Abdu et al., 2018). This may increase the effective *T*_a_ experienced by a small bird by about 12°C (Wolf, 2000), meaning birds in our study would be exposed to temperatures in excess of 57°C, which is well above the tolerance limit of most passerine birds (Abdu et al., 2018; McKechnie and Wolf, 2019). Therefore, zebra finches, like other desert birds, presumably face a trade-off between maintaining energy and water balance during extremely hot conditions and accessing food and water with exacerbated heat gain (Smit et al., 2016).

We found that zebra finches reduced their foraging activity for approximately 8 h in the middle of the day on hot compared to cool days. Finches balanced the reduced midday foraging on days with a high maximum *T*_a_ with increased foraging in the early cooler part of the day, and to a lesser extent in the evening (Figure 1). In contrast, birds foraged more consistently throughout the day during cooler weather. Previous studies of zebra finches at this site found that increased evening, rather than morning, foraging compensated for reduced midday foraging (Funghi et al., 2019), but for diurnal birds this strategy relies on sufficient environmental cooling in the evenings before dark for birds to forage. Environmental conditions are cooler in the early mornings than evenings (Figure 2), so anticipatory early morning foraging is likely a more favorable strategy during extremely hot conditions. However, birds must be able to anticipate reduced midday foraging to increase feeding in preparation for prohibitive daytime and evening conditions. Our data suggest that zebra finches were able to anticipate hot midday temperatures, presumably from overnight and early morning conditions, and increase their morning foraging accordingly. Similar pre-emptive responses to high maximum daytime *T*_a_ have been described for red kangaroos (*Osphranter rufus*) and western gray kangaroos (*Macropus fuliginosus*), whose nychthemeral reductions in *T*_b_ were greater in the early mornings of days with high maximum temperature than on days with lower maximum temperature, presumably facilitating heat storage later in the day (Brown and Dawson, 1977; Maloney et al., 2004). However, unlike kangaroos, which appear to pre-empt a favorable thermal response *via* an autonomous physiological feedback mechanism to environmental conditions (warming of the skin surface, resulting in distribution of core body heat to the cooler periphery and therefore a decrease in core *T*_b_; Maloney et al., 2004), our data suggest that finches behaviorally “plan” to withstand upcoming harsh environmental conditions.

### Mass, Energy, and Heat Balance

We found no difference in the zebra finches’ ability to maintain body mass over consecutive hot or cool days, despite evidence that reductions in foraging time and/or efficiency due to trade-offs with thermoregulatory behavior may significantly impact on energy balance of other birds, and that birds at high *T*_a_ may not be able to maintain body mass after consecutive warm days (du Plessis et al., 2012; Cunningham et al., 2015; Van de Ven, 2017). In fact, zebra finches maintained a higher body mass when it was hotter, and *T*_a_ was within or above the thermoneutral zone for the majority of each day (Figure 2). Although total foraging time of zebra finches during hot weather is less than during cool weather (Funghi et al., 2019), the reduced requirement for MHP means that total energetic requirements are lower, and a high-energy, highly digestible granivorous diet (MacMillen, 1990; Nagy, 2001) minimizes the fresh matter intake required for these birds compared to other species. Our data suggest that extremely hot weather did not place energetic constraints on the zebra finches by limiting feeding. Rather, cooler periods posed greater energetic challenges by requiring increased MHP to maintain a homeothermic *T*_b_ at *T*_a_ below thermoneutrality (Figure 2), and this may have been more challenging during a drought when natural food availability is low. Body size, diet, and foraging strategy presumably influence which bird species are most likely to experience energetic constraints from high temperatures (Edwards et al., 2015; Smit et al., 2016). Granivores, even those with food involving considerable handling time, may be able to meet their daily energetic requirements with “time to spare” and therefore do not need to forage for the entire daytime period. For example, cockatoos can meet their energetic returns by foraging for only 4–5 h a day (Cooper et al., 2002) and rest in shady locations for many hours, especially during summer (Noske, 1982; Cooper, personal observation). The finches in this study were provided with supplementary food to facilitate capture and data collection, which presumably assisted them in meeting their energy requirements more quickly than if they had to forage widely for all of their food. However, foraging behavior of individual birds suggests that they maintained natural foraging patterns when visiting the feeders (Funghi et al., 2019). A high BWC (75 vs. ~66%) and lack of breeding at the site also suggest poor body condition, and are evidence that the supplemental food provided was not excessive and did not compensate entirely for a scarcity of food as a consequence of the long-term drought.

Due to lower FMRs during hot periods, MWP was a smaller proportion of the total WTR of zebra finches, and therefore this difference (14% of WTR) had to be gained, presumably by increased drinking. Indeed, finches drank more frequently on hot compared to cool days (Figure 1), which maintained the same WTR. Measurements of physiological variables for zebra finches in the laboratory (Cooper et al,. in review) suggest that, despite their small body size, they use facultative hyperthermia to conserve water over the short term; a water savings of 9.17 mg g^−1^ h^−1^ measured for hyperthermic finches at 40°C (Cooper et al., in review) equates to a water savings of 57% of the mean water use by wild birds over 8 h. Finches can therefore delay dissipation of body heat until cooler environmental conditions facilitate non-evaporative heat loss, and this presumably is why birds during hot periods did not have a higher overall WTR than those during cool periods.

Despite overall higher drinking rates, distribution of visits by finches to water troughs decreased more in the middle of the day when it was hottest, and they increased their visits to water more in the early mornings and especially late afternoons (Figure 2), presumably preparing for, and then replenishing, water lost throughout the day. As they did for foraging, birds appear to be anticipating upcoming limitations on daytime drinking by drinking earlier, and more frequently early in the day, on extremely hot days. A high BWC may reduce the impact of short-term dehydration (e.g., as for amphibians; Hillman et al., 2009), and therefore limit reliance on visits to water during the heat of the day. If we assume that an 11.7-g bird with 66% BWC (Speakman, 1997) can lose ~11% of body mass as water before facing lethal dehydration (Wolf, 2000), then it can lose 1.287 ml of water (29% of their WTR) to retain 6.4 ml of body water. However, a bird of the same body mass starting with 75% body water can lose 2.34 ml of water to retain 6.4 ml of body water; they can lose 54% of their daily water turnover before risking lethal dehydration, extending the period birds can avoid drinking during periods of extreme environmental temperature. Indeed, finches did increase their drinking in the late afternoon on hot compared to cool days, presumably replenishing their body water store once the contribution of solar radiation to environmental temperature had decreased (Figure 2). Spinifexbirds (*Eremiornis carteri*; Ambrose et al., 1996) and Arabian babblers (*Turdoides squamiceps;* Anava et al., 2000) are other arid-habitat birds that have similarly high BWCs (up to 80%) that may also serve as a water store.

### Implications for Understanding High-Temperature Avian Mass Mortality

**Figure 3:**
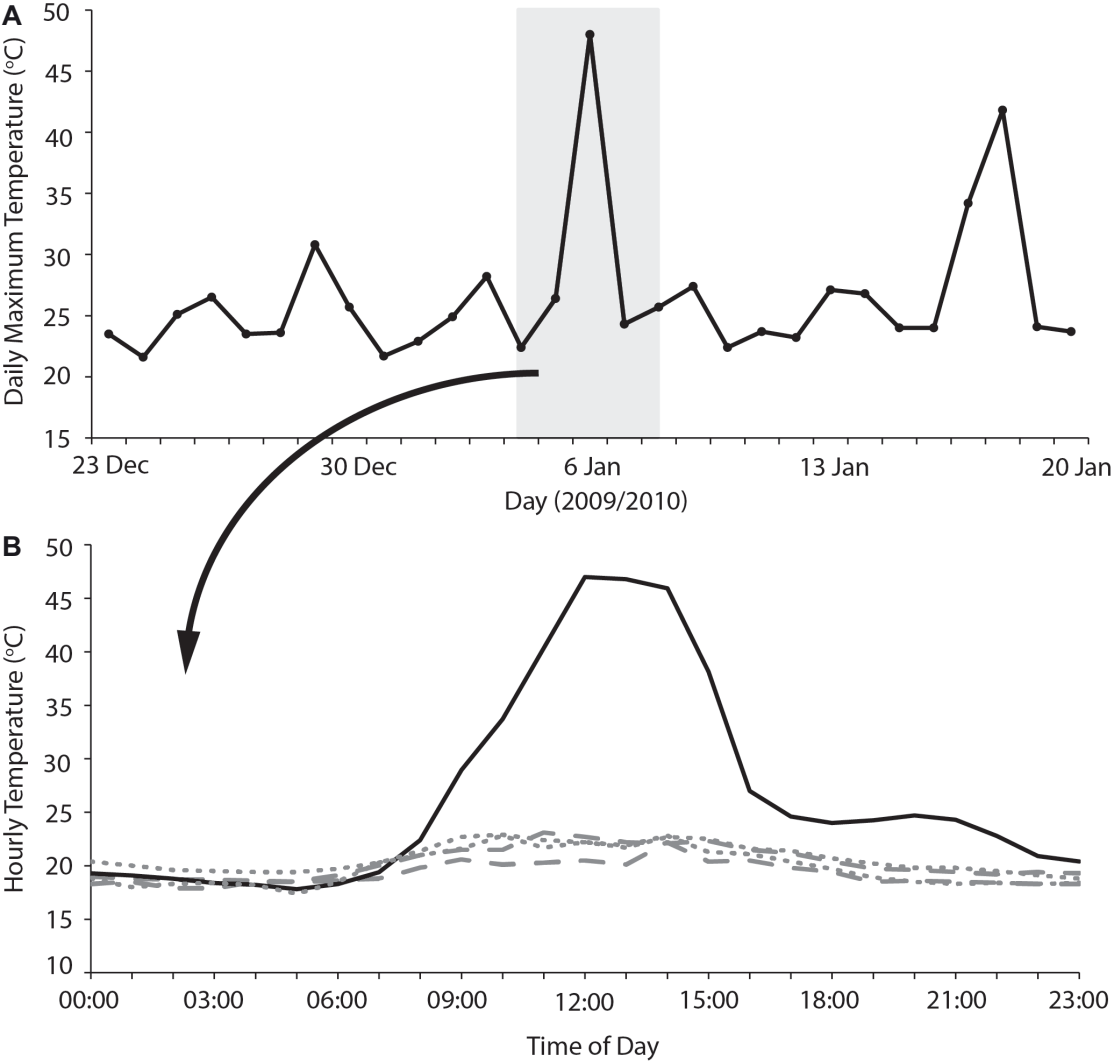
**(A)** Maximum daily temperatures for 2 weeks either side of an avian mass mortality event that occurred on the 6/2/2010 at Hopetoun, Western Australia. The gray shaded section indicates the 5 days where hourly temperature is plotted **(B)** for the 2 days before (dashes), 2 days after (dots), and the day of the avian mass mortality event (solid). Temperature data sourced from the Australian Bureau of Meteorology for Hopetoun North (station number 009961).

**Figure 4:**
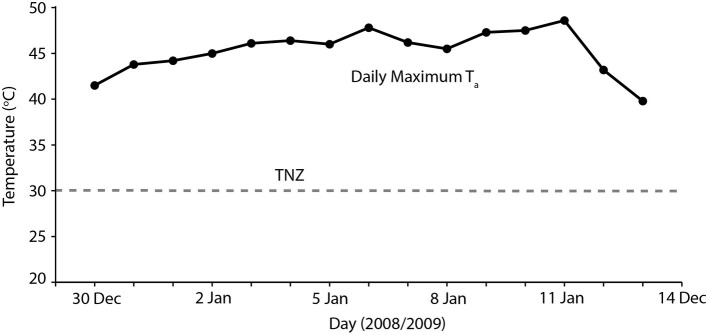
Maximum daily temperatures (black symbols) for 2 weeks during which time an avian mass mortality event occurred during January 2019, in the Pilbara region of Western Australia. The gray dashed line indicates thermoneutrality (TNZ; 30°C) for zebra finches (Calder, 1964; Cade et al., 1965). Temperature data sourced from the Australian Bureau of Meteorology for Gascoyne Junction (station number 006022).

Overall, our results indicate that heatwaves of 40–45°C maximum *T*_a_ can be accommodated by arid-adapted zebra finches without major impacts on energy and water turnover. Finches modified their behavior to avoid or limit activity during the most thermally challenging periods of the day and, together with a high BWC that presumably increased their body water store, were able to maintain body mass during a heatwave by pre-emptively feeding and drinking in preparation for hours of relative inactivity at high *T*_a_. We can apply this information to identify potential situations where avian mass mortality events can occur. Predicting upcoming periods of unfavorably high *T*_a_ may be essential for survival of extreme *T*_a_. One recently recorded avian mortality event, at Hopetoun, Western Australia, occurred when an extremely hot day (maximum *T*_a_ = 48°C) occurred between two much cooler days (maximum *T*_a_ = 24–26°C; Saunders et al., 2011; Figure 3), and following at least a fortnight of relatively cool summer maximum temperatures. Early morning temperatures on this day were indistinguishable from those of the preceding days; so, it is likely that birds experiencing this hot day did not “prepare” sufficiently in the cooler morning when feeding and more importantly drinking were possible, if the extremely high *T*_a_ was unexpected. The short-term predictability of heatwaves may be a determinate of avian survival. Another recent avian mass mortality event occurred at Overlander Roadhouse in the mid-west region of Western Australia (Birdlife Australia, 2014). In this case, maximum temperatures were very extreme, >40° for more than a fortnight, and ≥45°C for several consecutive days (Figure 4). Unfortunately, hourly temperature data are not available for a weather station close to this site, but it is likely that temperatures did not drop sufficiently during daylight hours over this prolonged heatwave to allow sufficient feeding or drinking to maintain energy and water stores. In addition, a previously good season with high winter and spring rainfall that facilitated breeding and subsequent large numbers of birds in the region (Birdlife Australia, 2014) presumably meant that birds were in good body condition, and may not have had the high lean BWC that we hypothesize may be critical for facilitating dehydration tolerance during the heat of the day.

To manage species and minimize biodiversity loss under the inevitable climatic changes that are occurring as a consequence of anthropogenic activity, we need a greater mechanistic understanding of how species physiologically accommodate environmental variability (Harris et al., 2018; Conradie et al., 2019). Our results reveal the consequences of short periods of high temperatures for an iconic desert bird in its natural habitat, and indicate the physiological and behavioral mechanisms by which zebra finches withstand extreme environmental conditions. This information has revealed that periods of low *T*_a_ during summer and particularly during droughts may pose greater energetic challenges than heatwaves for granivores. We also identify high BWC and predictable foraging limitations as potentially important elements for avian survival during periods of extreme heat. This information may assist in predicting and managing avian responses to periodic extreme weather events.

## Data Availability Statement

The datasets generated for this study are available on request to the corresponding author.

## Ethics Statement

The animal study was reviewed and approved by the Macquarie University and Curtin University Animal Ethics Committees (ARA 2017/024 and ARE2017-16).

## Author Contributions

CC and SG designed the study, provided equipment and logistical support, and obtained funding. CC and LH conducted the fieldwork. CC and PW carried out laboratory procedures and analyzed the data. CC drafted the manuscript. All authors edited the manuscript.

### Conflict of Interest

The authors declare that the research was conducted in the absence of any commercial or financial relationships that could be construed as a potential conflict of interest.
